# Intake of dietary fibre, red and processed meat and risk of late-onset Chronic Inflammatory Diseases: A prospective Danish study on the “diet, cancer and health” cohort

**DOI:** 10.7150/ijms.49314

**Published:** 2020-09-09

**Authors:** Katrine Hass Rubin, Nathalie Fogh Rasmussen, Inge Petersen, Tine Iskov Kopp, Egon Stenager, Melinda Magyari, Merete Lund Hetland, Anette Bygum, Bente Glintborg, Vibeke Andersen

**Affiliations:** 1 OPEN - Open Patient data Explorative Network, Department of Clinical Research, University of Southern Denmark, and Odense University Hospital, Odense Denmark.; 2 Focused Research Unit for Molecular Diagnostic and Clinical Research, IRS-Center Sonderjylland, Hospital of Southern Jutland, Aabenraa, Denmark.; 3 Danish Cancer Society Research Centre, Copenhagen, Denmark.; 4 The Danish Multiple Sclerosis Registry, Department of Neurology, Copenhagen University Hospital, Rigshospitalet, Copenhagen, Denmarkarch, University of Southern Denmark, Odense.; 5 MS clinic of Southern Jutland (Sønderborg, Esbjerg, Kolding) University Hospital of Southern Jutland, DK-6200 Aabenraa, Denmark.; 6 Department of Regional Health Research, University of Southern Denmark, DK-5000 Odense C, Denmark.; 7 National Institute of Public Health, University of Southern Denmark, Copenhagen, Denmark.; 8 The DANBIO registry and Copenhagen Center for Arthritis Research (COPECARE), Center for Rheumatology and Spine Diseases, Center of Head and Orthopaedics, Rigshospitalet, Glostrup, Denmark.; 9 Department of Clinical Medicine, Faculty of Health Sciences, University of Copenhagen, Copenhagen.; 10 Department of Clinical Genetics, Odense University Hospital, Odense, Denmark; University of Southern Denmark, Odense, Denmark.; 11 Research Unit of Dermato-Venerology, Institute of Clinical Research, University of Southern Denmark, Odense, Denmark.; 12 Institute of Clinical Research, University of Southern Denmark, Odense, Denmark.; 13 Institute of Molecular Medicine, University of Southern Denmark, Odense, Denmark.

**Keywords:** red meat, processed meat, dietary fibre, chronic inflammatory diseases

## Abstract

**Background:** Human and animal studies support the involvement of diet in the development of CID -chronic inflammatory diseases such as inflammatory bowel disease, psoriasis, rheumatoid arthritis, psoriatic arthritis, and multiple sclerosis.

**Objective:** This cohort study aimed to investigate the association between intake of fibre, red and processed meat, and occurrence of late-onset CID (50+ years of age) in the DCH: Danish Diet, Cancer and Health cohort. We hypothesised that risk of late-onset CID would be lower among those with high intake of fibre and/or low intake of meat compared to individuals with low fibre and/or high meat intake.

**Methods:** The DCH recruited 56,468 individuals, aged 50-64 years, between 1993 and 1997. At recruitment, diet intake was registered using food frequency questionnaires as well as lifestyle factors in 56,075 persons. Exposure variables were generated as sex-adjusted tertiles of fibre and meat (g/day). Development of CIDs was identified in national registries. Hazard ratios (HR) of late-onset CIDs (adjusted for age, sex, energy intake, alcohol, smoking, education, comorbidity, and civil status) were estimated for all three exposure variables.

**Results:** During follow-up of 1,123,754 years (median (Interquartile range) = 22.2 (20.1-23.1)), 1,758 (3.1%) participants developed at least one CID. The adjusted HRs for developing CID (*low fibre* 1.04 [0.89-1.22] and *medium fibre* 1.04 [0.91-1.18] (high fibre as reference), and *medium meat* 0.96 [0.86-1.09] and *high meat* 0.94 [0.82-1.07] (low meat as reference)) or the individual diseases were not statistically significant.

**Conclusion:** This large study did not support that a high intake of fibre and/or a low intake of meat had a high impact on the risk of late-onset CID.

## Introduction

Chronic inflammatory diseases (CIDs) include systemic diseases which primarily affect the intestine 1 and include inflammatory bowel disease (IBD) (Crohn's disease [CD] and ulcerative colitis [UC]), skin (psoriasis [PsO]), joints (chronic polyarthritis, including rheumatoid arthritis [RA], and psoriatic arthritis [PsA]) or the brain (multiple sclerosis [MS]).

Whereas the onset of CIDs may occur during the whole life-span 2-5 the risk of getting a CID late in life is high and increasing among the elderly 4, 6-10. Indeed, PsA and RA are commonly diagnosed after 60 years of age 6, 7. Age-adjusted (45 to 79 years of age) incidences (per 100,000 person-years) of the CIDs in Denmark are 6-10 for CD 2; 18-23 for UC 2; 200-225 for PsO 11; 28-35 for PsA 12; 4.4-7.5 for MS 7 and 47-81 for RA 13.

In recent years, a concept of CIDs sharing a “core disease signature” based in overlapping symptoms, genetics and environmental and lifestyle factors has arisen 1, 14. In particular, consumption of specific foods might increase (or reduce) inflammation and risk of CIDs - further attenuated by (lack of) exercise and other lifestyle factors including smoking 15-22. The “Western-style diet” is characterised by low fibre intake and high intakes of animal fat, red meat (from cows, pigs, goats, and sheep), sugar-sweetened drinks, fried food and lack of physical exercise. Several studies have reported this type of diet to be is associated with inflammatory responses 17, 23, 24. High red meat intake has also been associated with the development of inflammatory polyarthritis (including RA) 25. High fibre intake has been associated with a low risk of IBD in humans 26 and reduced intestinal inflammation in mice 27.

**Figure 1:**
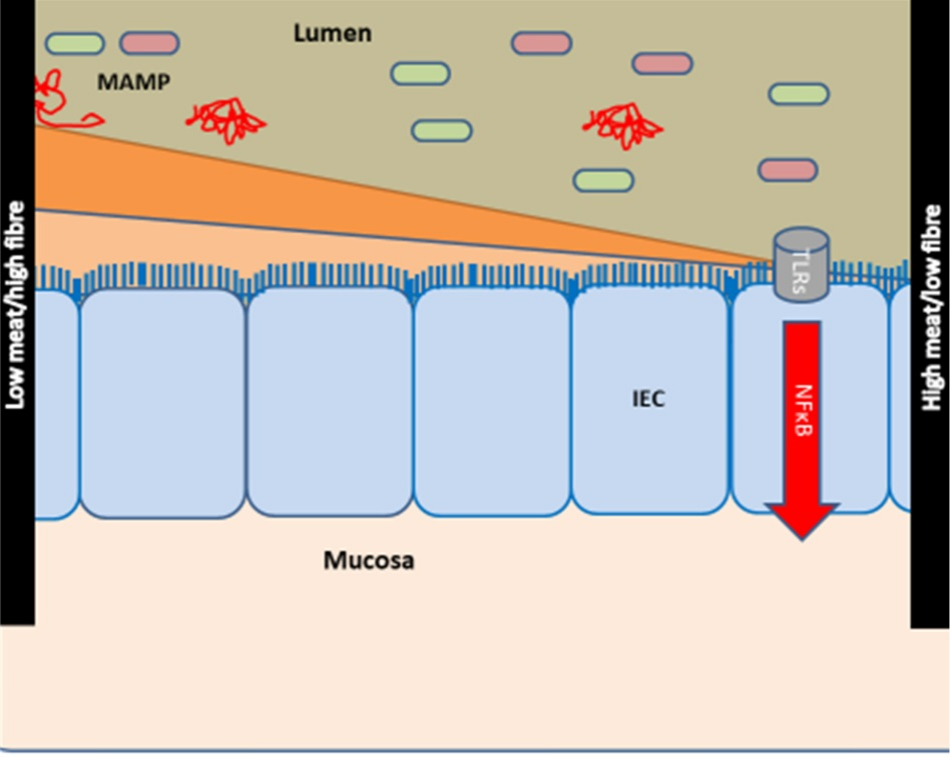
** Hypothesis of the effects of diet on the development of chronic inflammatory diseases.** The hypothesis is modified from 28, 29. Meat and fibre from the diet may affect the immune system 31-33 either directly or indirectly via e.g., the activity and composition of the gut microbiome 34, 35. The effect of low intake of fibre/high intake of red and processed meat is shown at right: low intake of fibre (which could otherwise serve as a nutrient for the microbes) causes microbial metabolism of mucus and decreases the intestinal mucus layer 36, 37. A high intake of red and processed meat renders the mucus layer penetrable to bacteria by reducing the disulphide bonds in the mucus network 29, 34. Thus, microbes may reach the epithelium 29, 38, 39 and activate the immune system 29, 40, 41. There is some support for such a mechanism in CIDs 29, including findings of high amounts of sulphate-reducing bacteria in IBD patients 29, 39, 42, the association of high fibre intake with a low risk of IBD among 170,776 participants from the prospective Nurses' Health Study I 26, 29, and the association of high intake of red meat and total protein and risk of developing inflammatory polyarthritis in the population-based prospective cohort of 25,630 participants from the European Prospective Investigation of Cancer in Norfolk 18, 25, 29. Abbreviations: CID: chronic inflammatory diseases, IBD: inflammatory bowel disease, IEC: intestinal epithelial cells, MAMP: Microbe-associated molecular patterns. Reproduced with permission from 30.

Based on the concept of a core disease signature, we provided the hypothesis that intake of high fibre/low red and processed meat may protect against inflammation across CIDs 28, 29. Thus, a diet high in meat and low in fibres may impact inflammation by affecting the composition of the gut microbiome 31 (**Figure 1**). In contrary to younger individuals, older individuals have been exposed to specific lifestyles and diets for many years. Therefore, this cohort study aimed to investigate the association between intake of fibre, red and processed meat and occurrence of CID among individuals 50+ years of age from the Diet, Cancer and Health (DCH) cohort (previously described in 30). We hypothesised that the risk of late-onset CID would be significantly lower among those with a high intake of fibre and/or a low intake of meat compared to individuals with low fibre and/or high meat intake 28.

## Methods and Materials

This cohort study was based on the DCH: Danish “Diet, Cancer and Health” cohort 43 and included prospective data from Danish nationwide health registries. A full study protocol is available 28.

### Study population

The DCH cohort was established between December 1993 and May 1997 and included a total of 56,468 individuals. The primary aim of the cohort was to investigate the association between specific dietary components and lifestyle with the risk of cancer and other chronic diseases 43. Criteria for inclusion were residency in Copenhagen or Aarhus area, age 50-64 years, born in Denmark and no previous cancer diagnosis in the Danish Cancer Registry. Upon inclusion (= baseline), the participants completed a lifestyle questionnaire and a FFQ - food frequency questionnaire 44, previously validated against two 7-day weighed diet records completed by 144 people 45. Furthermore, anthropometric measures were obtained by trained personnel.

**Figure 2:**
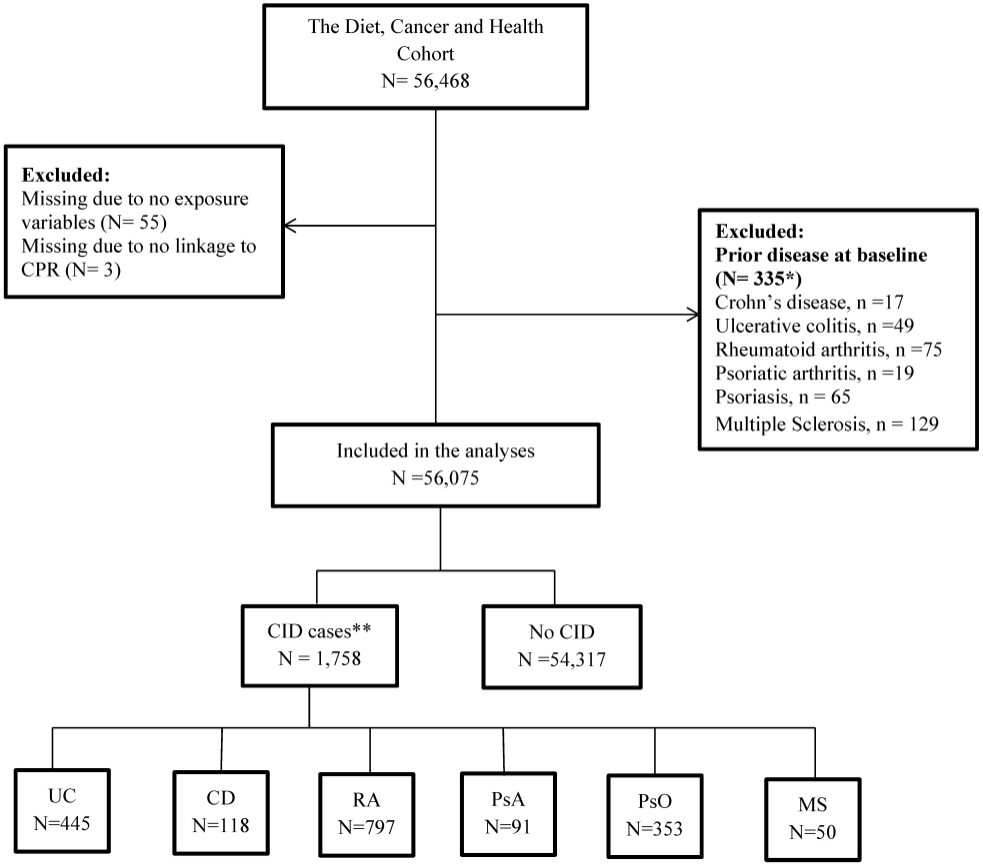
** Flow chart of the cohort.** *17 individuals had 2 CIDs, and 1 has 3 CIDs. Thus, the numbers do not add to 335; * 92 individuals had 2 CIDs, and 2 had 3 CIDs. Thus, numbers do not add to 1,758. Abbreviations: CID: Chronic Inflammatory Disease, UC: Colitis Ulcerosa, CD: Crohn's disease, RA: Rheumatoid Arthritis, PsA: Psoriatic Arthritis, PsO: Psoriasis, MS: Multiple Sclerosis.

In the present study, all participants from the DCH cohort were included except individuals with a prior diagnosis of CD, UC, PsO, PsA, RA or MS (**Table S1** in supplementary material) from a department with a relevant area of specialisation between 1977 and their date of entry into the DCH or DMSR (Danish Multiple Sclerosis Registry) cohorts. Some individuals were excluded because they did not deliver information about food intake or could not be linked with the DCRS: Danish Civil Registration System (**Figure 2**). The DCRS system is a mandatory nationwide registration system established in 1968 where all citizens are assigned a 10-digit unique identification number (CPR). The CPR is used as the key linkage across registers on an individual level 46.

### Outcome

The outcome was late-onset CID and included one or more of the following diseases: CD, UC, PsO, PsA, RA, or MS as identified in either the DNPR: Danish National Patient Registry 47 or the DMSR: Danish Multiple Sclerosis Registry 48.

The DNPR is a mandatory nationwide health register established in 1977, which comprises of information about in- and outpatient-contacts with Danish hospitals 47. In this study, the DNPR was used to identify patients diagnosed with CIDs. Furthermore, information on the departments with relevant areas of specialization was obtained from the DNPR.

The DMSR was established in 1956 and includes all Danish citizens who have been diagnosed with MS. The register also includes patients with a MS or MS-related diagnosis from the DNPR 49. The completeness of the registry is estimated to be about 90-95% and the validity is around 94% 48.

Outcomes were identified according to the following criteria 1) receiving a relevant A-diagnosis (ICD8 or ICD10) in years primo 1977 to ultimo 2018 in DNPR (except MS) from a department with a relevant area of specialization followed by at least one additional registration in DNPR (inpatient or outpatient visit) related to the first diagnosis within 180 days (except RA where 90 days was used as the cutpoint following a previous validity study 50), or 2) having a MS diagnosis registered in the DMSR. Date of diagnosis was set to the first date of the first relevant A-diagnosis, except for MS where only the year of diagnosis was available. Thus, for MS the date was set to June 30 from the year the MS diagnosis was received from the DMSR registry (**Supplementary Table S1**). A few individuals had more than one CID diagnosis (92 had two and 2 had three CID diseases). For these individuals, only the first diagnosis was included in the analyses.

Information on civil status, death and immigration during follow up was retrieved from the DCRS.

### Exposure assessment, fibre and meat

In the DCH cohort, the participants' usual intake of food and beverages during the past 12 months was collected from the validated 192-item FFQ including 12 possible response categories ranging from “never” to “eight times or more per day”. The participants' average food and nutrient intakes were calculated using FoodCalc 51 which uses gender-specific portion sizes and the reported frequency of portions consumed to calculate intake in grams per day (g/day) 45.

Definition of meat variables; the sum of red meat (g/day) including fresh beef, veal, pork, lamb and offal (heart and liver) and processed meat including various kinds of cold cuts, sausages, liver pate and bacon items which had undergone processing such as smoking, salting or curing.

Definition of dietary fibre intake; total fibre intake was calculated by multiplying the frequency of consumption of relevant foods (i.e., fruit, vegetables, cereals, and leguminous fruit) by their fibre content as determined from national databases of food content.

Exposure was defined as gender-specific tertiles of intake of dietary fibre and meat. Furthermore, the tertiles of fibre and meat intake were combined into a categorical exposure variable used in a previous Danish prospective, case-cohort study using the DCH cohort 52. The variable included four exposure groups: a low-risk group, two medium-risk groups and a high-risk group (see **Table 1** for details).

### Covariates

The analyses were adjusted for potential sociodemographic, lifestyle and health-related confounding covariates.

#### Sociodemographic covariates

Sex and age, cohabitation and years of highest achieved education at the time of response to the DCH questionnaire (baseline). Cohabitation was retrieved from the DCRS and was categorized as living together (married and living together), or living alone (single, divorced or widow/widower). Years of education was categorized into “<8years”, “8-9years”, and “>9 years”.

#### Lifestyle covariates

Total energy intake (MJ/day) obtained at baseline from the DCH survey, alcohol intake (g/day), and smoking status (never, former, current).

#### Health-related covariates

Quan's updated version of Charlson's comorbidity index (CCI) (registered in DNPR 10 years before the date of interview) was estimated to classify with/without comorbidity 10-years before participation in the DCH survey 53.

### Statistical methods

Baseline characteristics were summarised and stratified by the occurrence of CID (yes/no) in the follow-up period using medians and 25^th^ and 75^th^ percentiles for continuous variables. Nonparametric tests were used to test for differences between groups. Categorical variables were presented as frequencies, and differences between groups evaluated by χ^2^-square test.

Incidence rates (IRs) of late-onset CID per 1,000 person-years were calculated stratified for tertiles of intake of fibre and meat as well as the four exposure groups of combined tertiles of fibre and meat intake.

Furthermore, unadjusted as well as covariate-adjusted (age, sex, energy intake, alcohol, smoking, education comorbidity, and civil status) Cox regression analyses estimating the hazard ratio (HR) of late-onset CID were performed. The proportional hazard assumption was tested using Schoenfeld residuals 54, and sensitivity analyses modelling the time-varying effect of covariates that did not fulfil the proportional hazard assumption, were performed.

Additional sensitivity analyses were conducted estimating HRs for the individual disease groups, and sensitivity analyses based on subsets of the sample excluding individuals who were identified as suffering from a CID within 0.5, 1, and 5 years after filling out the questionnaire. Lastly a sensitivity analysis was conducted estimating HR for CID restricting the follow-up period to 10 years.

All analyses were carried out using STATA version 15 55. For all tests, a *p*-value below 0.05 was considered statistically significant.

### Ethical approval

The study was approved by the Danish Data Protection Agency, and the patient consent was obtained before entering the DCH cohort. The study ''Diet, Cancer and Health'' has been approved by the relevant Scientific Committees and the Danish Data Protection Agency. According to Danish law, ethical approval is not required for register-based studies. All personal level data were pseudo-anonymized and handled at secure servers at the Danish Health Authority.

## Results

Among 56,468 individuals recruited for the DCH cohort, 56,075 individuals were included (for details see flow chart in** Figure 2**).

**Table 2** presents the summary statistics of covariates stratified for CID outcome. CID cases compared to non-cases were more frequently females, had shorter education, less energy intake, lower intake of fibres and meat, higher frequency of smoking, less alcohol use, and more comorbidity. No statistically significant differences were observed for age, BMI and physical activity, borderline statistical significance was reached for marital status (*p*=0.05).

Incidence rates per 1,000 person-years (IRs) of CID are reported in **Table 3**. Across tertiles of fibre intake, the IR of CID was lowest among patients with the highest intake of fibres (IR= 1.47 and highest among patients with the lowest intake of fibres (IR= 1.66). In the middle tertile, the incidence rate was 1.57, demonstrating a trend towards a higher risk of CID with decreasing intake of fibres. No trend in the incidence rates was seen across tertiles of meat intake (1.58, 1.56, 1.56, respectively). For the combined exposure, the estimated IR ranged from 1.51 (low-risk group) to 1.65 (high-risk group), with the medium-risk group A virtually identical (1.53) to the low-risk group (supporting the importance of fibre compared to meat), and similarly the medium-risk group B was virtually identical with the high-risk group, IR= 1.66).

**Figure 3:**
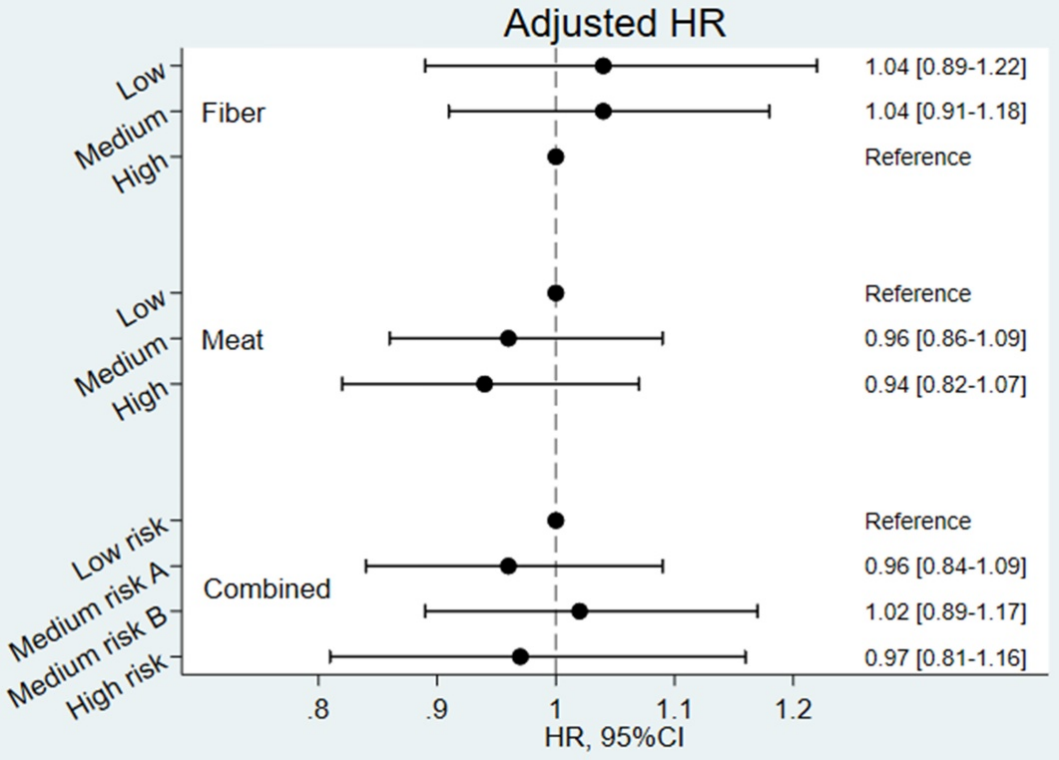
** Covariate adjusted Hazard Ratios (HR) for developing Chronic Inflammatory Disease (CID) (N=54,303).** Combined exposure: Low risk: low/medium intake of meat and medium/high intake of fibres; Medium risk A: high intake of meat and medium/high intake of fibres; Medium risk B low/medium intake of meat and low intake of fibres; High risk: high intake of meat and low intake of fibres. Model adjusted for age, sex, energy (MJ/day), alcohol intake (g/day), smoking status (never, former, current), education (<8years, 8-9years, >9years), civil status (Living alone, cohabiting), and co-morbidity (Charlson comorbidity index 1+ vs 0).

Unadjusted and covariate-adjusted Hazard Ratios for developing CID are shown in **Figures S1 and Figure 3**, respectively. The Cox analyses confirmed the results from the incidence rates (i.e. none of the Hazard Ratios were statistically significant). In the unadjusted analyses, tendencies towards a higher risk among participants with a low fibre intake were observed. However, in the adjusted analyses these tendencies were weakened and completely vanished for the combined exposure variable. The adjusted Cox-analyses revealed that female sex, smoking and comorbidity were significantly associated with a risk of developing CID in late life (results shown in Supplementary **Table S2**).

Sensitivity analyses demonstrated that education level and comorbidity did not meet the requirements for proportional hazard assumptions; however, modelling time-varying effects of these variables did not change the HRs for the exposure variables (results not shown). Similarly, repeating the analyses on samples of individuals whose CID diagnosis were identified with a 0.5, 1, and 5 years delay after the interview did not change the results of the study (**Table S3**). The sensitivity analyses of the individual disease groups demonstrated few statistically significant results in the unadjusted analyses (**Table S4**) of which none maintained significance in the adjusted analyses (**Table 4**). Restricting the follow-up period to 10 years did not change the overall results of HR for developing CID (results not shown).

## Discussion

In this large prospective study of 56,075 Danish middle-aged men and women, we identified 1,758 individuals with at least one CID diagnosis during a median follow-up period of 22.2 years. In our published pre-study protocol 28, we hypothesized that individuals with a high intake of fibre and a low intake of red and processed meat would have a lower risk of late-onset CID compared to individuals with a low intake of fibre and a high intake of meat. We found a trend towards a lower risk of CID among patients with the highest intake of fibres. However, it was only present in univariate and not adjusted analysis of CID overall or the individual diseases IBD, RA, PsA, PsO or MS. Furthermore, we found no effect of meat or combined meat and fibre exposure. The lack of association for fibre in the adjusted analyses was primarily explained by sex, smoking and comorbidity. This is not surprising since previous studies have demonstrated a higher risk of chronic inflammatory diseases in females, smokers, and individuals with comorbidities 19, 56, 57.

This is the first study to report associations between fibre and red and processed meat intake and risk of CID that combines different CIDs in one outcome measure, and the first study (to our best knowledge) to report on these exposures in relation to MS, PsO, and PsA. Two prospective studies investigated the intake of dietary fibres and the risk of developing IBD. The US Nurses' Health Study (NHS) 26 enrolled participants at 25-55 years of age and followed them for 26 years. A similar number of cases compared with this study were identified, (269 CD and 338 UC cases). In the US Nurses study, the highest quintile of dietary fibre [median 24.3 g/day] was associated with a 41% lower risk of CD compared to the lowest quintile of fibre intake (multivariate HR for CD, 0.59; 95% confidence interval, 0.39-0.90) 26. In a large European study of 401,326 men and women aged 20-80 years (EPIC-IBD), 104 and 221 cases developed CD and UC, respectively. No statistically significant associations between intake of fibre and disease were found 58. In addition, two prospective studies investigated the intake of red and processed meat and the risk of developing RA. A Swedish study of 35,600 women in a similar age group as the present study identified 368 cases that developed RA. No association with the intake of meat and risk of RA was found whereas the impact of fibre was not included 59. In contrast, the EPIC-Norfolk study (aged 45-75), reported that subjects in the highest tertile for red and processed meat consumption (>88 g red meat/day) were at an increased risk of inflammatory polyarthritis (OR 2.3, 95% CI 1.1-4.9) compared to subjects in the tertile with the lowest level of consumption (<49 g/day) 25. There are several differences between these two studies that may account for the different results such as differences in age groups, study populations and diet and lifestyle factors not accounted for, that may impact the results.

This study has several strengths, firstly the use of a prospective cohort and nationwide registries, which reduced the possibility of selection bias. The linkage to the Danish health registries ensured almost complete follow-up of the study population, as the Danish health registries are considered comprehensive with high validity 47, 60. Secondly, the use of a very restrictive criteria for defining late-onset CID cases requiring that cases fulfilled diagnostic criteria according to both type, place and time of diagnosis or were registered in the DMSR. This approach ensured high specificity and that a high proportion of the identified cases had late-onset CID. Validation studies have been performed on the overall quality of the administrative and clinical data in the DNPR with medical records as a reference, and the quality of the individual variables in the register including the accuracy of the ICD coding 47. Although the overall quality of the information in the DNPR is considered to be high, the validity of specific diagnoses may be more uncertain 47. Thirdly, the prospective study design with the collection of dietary and lifestyle factors before diagnosis eliminates the risk of recall bias. A fourth advantage is diverse and high intake of meat and fibre in the cohort enabled investigations of the effects of diet and therefore the prospective “Diet, Cancer and Health” cohort has been proven suitable to detect effects of meat and fibre intake 61-64. A fifth strength of this study is the consideration that individuals diagnosed with CID within 0.5, 1, and 5 years after filling out the questionnaire may have biased the results if they had systematically different exposure levels than the other late-onset CID cases. However, the sensitivity analysis did not indicate bias. And lastly, this study's inclusion of relevant life-style factors further strengthens our results.

The study has some limitations. Firstly, causality between exposure and outcome cannot be established in an observational study. Secondly, the diet was only assessed once, and it can be questioned whether the dietary intake at baseline was representative for the intake during follow-up. Also, limitations according to the DCH questionnaire and the self-reported information should be considered e.g. changes in the diet and lifestyles during the last 25 years. Changes in dietary and lifestyle habits during follow-up, if present, will result in a lower power to detect real differences between cases and comparisons. However, the DCH questionnaire has been demonstrated to be a reliable and valid tool 45 and has been used in several other studies during the last 5-10 years 52, 62, 65. Thirdly, another disadvantage of the study is the limited power due to the low number of CID incidences, pronounced mostly in the specific autoimmune disease stratified analyses. A fourth limitation is the disease groups may be heterogeneous regarding dietary and lifestyle factors, and the analyses may not have captured these differences (residual confounding). The fifth limitation is that exposure groups in our study are crude estimates of dietary intake of meat and fibres, and are subject to heterogeneity within the groups. Finally, the CIDs included in this study may be too heterogeneous to be analysed as one group.

## Conclusion

Although this study shows trends towards a higher risk of late-onset CID with a diet which is lower in fibre intake, and no significant risk of late-onset CID in relation to meat intake, this large study did not support that a high intake of fibre and/or a low intake of meat had a high impact on the risk of late-onset CID. Large, prospective cohorts with detailed dietary information are needed for future studies.

## Supplementary Material

Supplementary figures and tables.

## Figures and Tables

**Table 1:**
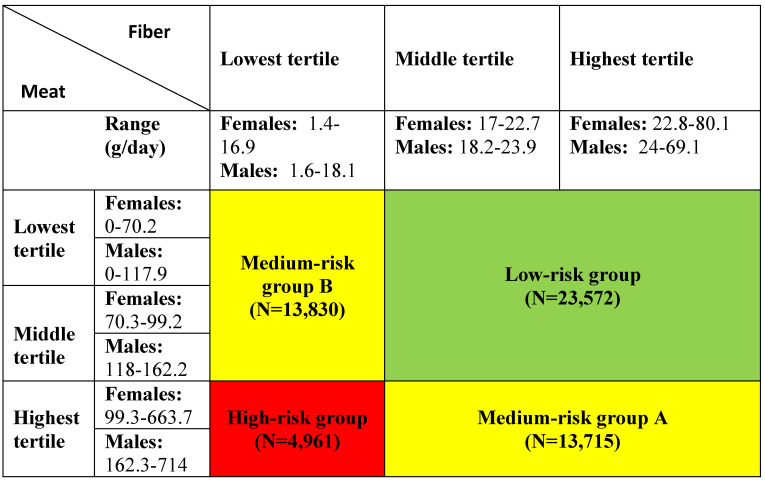
Definition of exposure variables

**Table 2:** Baseline characteristics of the study population by the occurrence of Chronic Inflammatory Disease

	Chronic inflammatory disease during follow-up		
	No. (N=54,317)	Yes (N=1,758)	*p*-value
**Demographics**			
Age, years*^a^*	56.2 (52.8;60.4)	56.3 (52.7;60.4)	0.75
Gender*^b^*			<0.001
Male	26,100 (48.1)	675 (38.4)	
***Marital status^b^***			0.05
Living alone	14,151 (26.1)	503 (28.6)	
Living together	38,535 (70.9)	1,209 (68.8)	
Missing	1,631 (3.0)	46 (2.6)	
***Education (years)^b^***			0.01
<8	17,882 (32.9)	612 (34.8)	
8-9	24,974 (46.0)	814 (46.3)	
>9	11,400 (21.0)	327 (18.6)	
Missing	61 (0.1)	5 (0.3)	
**Lifestyle**			
Energy (MJ/day)*^a^*	9.5 (7.9; 11.4)	9.2 (7.7; 11.1)	0.001
Total dietary fibre intake (g/day)*^a^*	20.3 (16.0; 25.2)	19.8 (15.9; 24.7)	0.02
Meat (g/day)*^a^*	106.4 (76.3; 146.3)	100.9 (71.4; 137.4)	0.001
Physical activity (MET-score)*^a^*	56.5 (37.0; 85.0)	55.0 (36.0; 86.5)	0.52
Alcohol (g/day)*^a^*	13.0 (6.0; 31.2)	11.4 (3.4; 27.4)	0.001
**Smoking*^b^***			<0.001
Never	19,166 (35.3)	481 (27.4)	
Former	15,626 (28.8)	495 (28.2)	
Current	19,456 (35.8)	779 (44.3)	
Missing	69 (0.1)	3 (0.2)	
**BMI (kg/m^2^)*^b^***			0.35
<25.0	23,485 (43.2)	740 (42.1)	
≥25.0	30,800 (56.7)	1,016 (57.8)	
Missing	32 (0.1)	2 (0.1)	
**Comorbidity (Charlson score)*^b^***			<0.001
0	52,676 (97.0)	1,590 (90.4)	
1+	1,641 (3.0)	168 (9.6)	

*a*, Median (interquartile range); *b*, Numbers (percentages).

**Table 3:** Incidence rates per 1,000 person-years of Chronic Inflammatory Diseases

	N	No. of cases	IR	95% CI
**Dietary fibre - tertiles of g/day**				
Lowest	18,791	610	1.66	1.53-1.80
Middle	18,710	591	1.57	1.44-1.70
Highest	18,574	557	1.47	1.35-1.60
**Meat - tertiles of g/day**				
Lowest	18,716	596	1.58	1.45-1.71
Middle	18,686	583	1.56	1.43-1.69
Highest	18,673	579	1.56	1.44-1.69
Combined exposure - combinations of tertiles of meat and fibre intake^*^				
Low risk group *^a^*	23,572	728	1.51	1.40-1.62
Medium risk group A*^b^*	13,712	420	1.53	1.39-1.68
Medium risk group B*^c^*	13,830	451	1.66	1.52-1.83
High risk group *^d^*	4,961	159	1.65	1.41-1.93

* Combined exposure: *a,* low/medium intake of meat and medium/high intake of fibres; *b*, high intake of meat and medium/high intake of fibres; *c*, low/medium intake of meat and low intake of fibres; d high intake of meat and low intake of fibres.

**Table 4:** Covariate-adjusted Hazard Ratios (HR [95%CI]) for Inflammatory Bowel Disease, Rheumatoid Arthritis, Psoriasis, Psoriatic arthritis, and Multiple sclerosis

Exposure	Inflammatory bowel disease (IBD)	Rheumatoid arthritis (RA)	Psoriasis (PsO)	Psoriatic arthritis (PsA)	Multiple sclerosis (MS)
	Adjusted^**^ HR (95%CI) (N=54,567)	Adjusted^**^ HR (95%CI) (N=54,558)	Adjusted^**^ HR (95%CI) (N=54,566)	Adjusted^**^ HR (95%CI) (N=54,613)	Adjusted^**^ HR (95%CI) (N=54,499)
Number (%) of cases	542 (1.0)	799 (1.4)	357 (0.6)	97 (0.2)	50 (0.1)
**Dietary fibre - tertiles of g/day**					
Lowest	1.20 (0.91;1.60)	0.95 (0.75;1.19)	0.97 (0.69;1.38)	0.61 (0.32;1.16)	1.42 (0.56;3.62)
Middle	1.16 (0.92;1.47)	1.00 (0.83;1.21)	0.96 (0.72;1.29)	0.64 (0.38;1.10)	1.29 (0.58;2.83)
Highest	1 (ref)	1 (ref)	1 (ref)	1 (ref)	1 (ref)
**Meat - tertiles of g/day**					
Lowest	1 (ref)	1 (ref)	1 (ref)	1 (ref)	1 (ref)
Middle	1.07 (0.86;1.32)	0.86 (0.72;1.02)	1.05 (0.80;1.39)	0.97 (0.58;1.60)	1.09 (0.54;2.22)
Highest	0.95 (0.75;1.21)	0.84 (0.69;1.02)	1.29 (0.97;1.72)	1.06 (0.61;1.83)	1.27 (0.59;2.71)
Combined exposure - combinations of tertiles of meat and fibre intake^*^					
Low risk*^a^*	1 (ref)	1 (ref)	1 (ref)	1 (ref)	1 (ref)
Medium risk A*^b^*	1.01 (0.80;1.28)	0.86 (0.71;1.05)	1.22 (0.92;1.64)	0.99 (0.57;1.73)	1.27 (0.58;2.77)
Medium risk B*^c^*	1.19 (0.94;1.52)	0.91 (0.74;1.11)	0.95 (0.70;1.30)	0.75 (0.42;1.36)	1.21 (0.54;2.70)
High risk*^d^*	0.83 (0.58;1.19)	0.95 (0.73;1.23)	1.25 (0.87;1.79)	1.04 (0.51;2.12)	1.29 (0.46;3.57)

*Combined exposure: *a*, low/medium intake of meat and medium/high intake of fibres; *b*, high intake of meat and medium/high intake of fibres; *c*, low/medium intake of meat and low intake of fibres; *d,* high intake of meat and low intake of fibres. **Model adjusted for age, sex, energy (MJ/day), alcohol intake (g/day), smoking status (never, former, current), education (<8years, 8-9years, >9years), civil status (Living alone, cohabiting), and co-morbidity (Charlson comorbidity index 1+ vs 0).
