# Supplementary material for: Intake of dietary fibre, red and processed meat and risk of late-onset Chronic Inflammatory Diseases: A prospective Danish study on the “diet, cancer and health” cohort
Source: Int J Med Sci. 2020 Sep 9;17(16):2487–95. doi: 10.7150/ijms.49314 (PMC7532485; doi:10.7150/ijms.49314)
Supplement: Supplementary file 1 — Supplementary figures and tables. [file ijmsv17p2487s1.pdf]

Supplemental tables and figure:

Supplementary Table S1: Specification of outcome CIDs with associated diagnostic- and treatment codes

	**Specialized hospital unit**	**ICD8**	**ICD10**	**Comment**
Chrohn's Disease (CD)	Surgical Gastroenterology, Medical Gastroenterology, Internal medicine	563.00, 563.91, 563.02, 563.08, 563.09	DK50*	Date of diagnosis: First relevant A-diagnosis from specialized hospital department, followed by minimum one diagnosis or ambulant visit within 180 days (RA: 90days)
Ulcerative colitis (UC)	Surgical Gastroenterology, Medical Gastroenterology, Internal medicine	563.19, 563.99, 569.04	DK51*	
Rheumatoid Arthritis (RA)	Rheumatology, Internal medicine	712.19, 712.29, 712.39, 712.59	DM059, DM060, DM068, DM069	
Psoriatic Arthritis (PsA)	Rheumatology, Internal medicine	696.09	DM071, DM072, DM073, DM090, DM468	
Psoriasis (PsO)	Dermato-venerology, Internal medicine	696.10, 696.19	DL40*	
Multiple Sclerosis (MS)	---	Nationwide, population-based MS registry	Nationwide, population-based MS registry	Year of diagnosis is given. Date set to mid-year (i.e. yyyy0630)
Inflammatory Bowel Disease (IBD)	---	CD or UC	CD or UC	Date of diagnosis: Minimum of the dates for CD and UC
Chronic Inflammatory disease (CID)	---	one of the above mentioned	one of the above mentioned	Date of diagnosis: Minimum of the above dates

*including all subcodes

Supplementary Table S2: Hazard ratios (HR, 95%CI, and p-values) of risk of developing late-onset CID from multiple Cox-regression. Main exposure variable is fibre intake.

Covariate	HR	95% CI	p-value
**Fibre**			
**Low**	1.04	0.89-1.22	0.60
**Medium**	1.04	0.91-1.18	0.58
**High**	1 (reference)	---	---
**Age**	1.00	0.99-1.02	0.43
**Sex**			
**Male**	0.70	0.62-0.78	<0.001
**Female**	1 (reference)	---	---
**Energy intake**	1.00	0.98-1.03	0.98
**Alcohol**	1.00	0.99-1.00	0.12
**Smoking**			
**Never**	1 (reference)	---	---
**Former smoking**	1.39	1.22-1.58	<0.001
**Current smoker**	1.82	1.61-2.04	<0.001
**Education**			
**Less than 8 years**	1 (reference)	---	---
**8-9 years**	0.97	0.88-1.09	0.64
**10+ years**	0.92	0.80-1.05	0.22
**Comorbidity**			
**No**	1 (reference)	---	---
**Yes**	3.69	3.13-4.34	<0.001
**Married/cohabitant**			
**No**	1 (reference)	---	---
**Yes**	0.99	0.89-1.10	0.88

Supplementary Table S3: Unadjusted and covariate-adjusted Hazard Ratios (HR (95%CI)) from Cox-analyses on participants identified with a 0.5, 1, and 5 year's delay after interview

Exposure	0.5 year delay		1 year delay		5 year's delay	
	Unadjusted HR (95%CI) (N=55,959)	Adjusted ** HR (95%CI) (N=54,194)	Unadjusted HR (95%CI) (N=55,829)	Adjusted ** HR (95%CI) (N=54,072)	Unadjusted HR (95%CI) (N=54,059)	Adjusted ** HR (95%CI) (N=52,435)
**Number (%) of cases**	1,719 (3.1)		1,676 (3.0)		1,238(2.3)	
**Dietary fibre - tertiles of g/day**						
Lowest	1.10 (0.96;1.26)	0.99 (0.83;1.19)	1.12 (1.00;1.26)	1.02 (0.87;1.20)	1.10 (0.96;1.26)	0.99 (0.83;1.19)
Middle	1.04 (0.90;1.19)	1.00 (0.86;1.16)	1.05 (0.94;1.19)	1.02 (0.90;1.17)	1.04 (0.90;1.19)	1.00 (0.86;1.16)
Highest	1 (ref)	1 (ref)	1 (ref)	1 (ref)	1 (ref)	1 (ref)
**Meat - tertiles of g/day**						
Lowest	1 (ref)	1 (ref)	1 (ref)	1 (ref)	1 (ref)	1 (ref)
Middle	1.06 (0.92;1.21)	1.03 (0.89;1.18)	0.99 (0.88;1.12)	0.97 (0.86;1.10)	1.06 (0.92;1.21)	1.03 (0.89;1.18)
Highest	1.05 (0.91;1.20)	0.99 (0.85;1.16)	1.01 (0.90;1.13)	0.96 (0.84;1.10)	1.05 (0.91;1.20)	0.99 (0.85;1.16)
**Combined exposure - combinations of tertiles of meat and fibre intake***						
Low risk [a]	1 (ref)	1 (ref)	1 (ref)	1 (ref)	1 (ref)	1 (ref)
Medium risk A [b]	0.97 (0.84;1.12)	0.92 (0.79;1.08)	1.01 (0.90;1.15)	0.96 (0.84;1.10)	0.97 (0.84;1.12)	0.92 (0.79;1.08)
Medium risk B [c]	1.03 (0.89;1.18)	0.94 (0.80;1.11)	1.08 (0.96;1.22)	1.00 (0.87;1.15)	1.03 (0.89;1.18)	0.94 (0.80;1.11)
High risk [d]	1.20 (0.98;1.46)	1.05 (0.86;1.29)	1.13 (0.95;1.34)	1.00 (0.83;1.19)	1.20 (0.98;1.46)	1.05 (0.86;1.29)

***Combined exposure:** [a] low/medium intake of meat and medium/high intake of fibres; [b] high intake of meat and medium/high intake of fibres; [c] low/medium intake of meat and low intake of fibres; [d] high intake of meat and low intake of fibres.

** Model adjusted for age, sex, energy (MJ/day), alcohol intake (g/day), smoking status (never, former, current), education (<8years, 8-9years, >9years), civil status (Living alone, cohabiting), and co-morbidity (Charlson comorbidity index 1+vs 0).

Supplementary Table S4: Unadjusted Hazard Ratios (HR (95%CI)) for Inflammatory Bowel Disease (IBD), Rheumatoid Arthritis (RA), Psoriasis (PsO), Psoriatic arthritis (PsA) and Multiple sclerosis (MS).

**Exposure**	**Inflammatory bowel disease (IBD)**<br><br>**Unadjusted HR (95%CI) (N=56,351)**	**Rheumatoid arthritis (RA)**<br><br>**Unadjusted HR (95%CI) (N=56,337)**	**Psoriasis (PsO)**<br><br>**Unadjusted HR (95%CI) (N=56,349)**	**Psoriatic arthritis (PsA)**<br><br>**Unadjusted HR (95%CI) (N=56,397)**	**Multiple sclerosis (MS)**<br><br>**Unadjusted HR (95%CI) (N=56,281)**
**Number (%) of cases**	542 (1.0)	799 (1.4)	357 (0.6)	97 (0.2)	50 (0.1)
**Dietary fibre - tertiles of g/day**					
Lowest	1.12 (0.91;1.38)	1.09 (0.92;1.29)	1.23 (0.95;1.59)	0.83 (0.52;1.34)	1.48 (0.73;3.00)
Middle	1.11 (0.90;1.36)	1.05 (0.89;1.25)	1.08 (0.83;1.40)	0.74 (0.45;1.20)	1.38 (0.68;2.82)
Highest	1 (ref)	1 (ref)	1 (ref)	1 (ref)	1 (ref)
**Meat - tertiles of g/day**					
Lowest	1 (ref)	1 (ref)	1 (ref)	1 (ref)	1 (ref)
Middle	1.09 (0.89;1.34)	0.87 (0.74;1.03)	1.10 (0.85;1.44)	0.97 (0.59;1.60)	0.95 (0.48;1.87)
Highest	0.99 (0.80;1.22)	0.91 (0.77;1.07)	1.33 (1.03;1.71)	1.08 (0.67;1.75)	1.01 (0.52;1.98)
**Combined exposure - combinations of tertiles of meat and fibre intake***					
Low risk [a]	1 (ref)	1 (ref)	1 (ref)	1 (ref)	1 (ref)
Medium risk A [b]	1.05 (0.85;1.31)	0.93 (0.77;1.11)	1.27 (0.98;1.65)	1.00 (0.60;1.65)	1.10 (0.53;2.26)
Medium risk B [c]	1.17 (0.95;1.44)	1.01 (0.85;1.20)	1.20 (0.92;1.57)	0.88 (0.52;1.48)	1.29 (0.65;2.57)
High risk [d]	0.87 (0.62;1.22)	1.10 (0.86;1.41)	1.60 (1.13;1.26)	1.18 (0.59;2.35)	1.29 (0.48;3.44)

***Combined exposure:** [a] low/medium intake of meat and medium/high intake of fibres; [b] high intake of meat and medium/high intake of fibres; [c] low/medium intake of meat and low intake of fibres; [d] high intake of meat and low intake of fibres.

Supplemental Figure S1: Unadjusted Hazard Ratios (HR) for developing Chronic Inflammatory Disease (CID) (N=56,075)

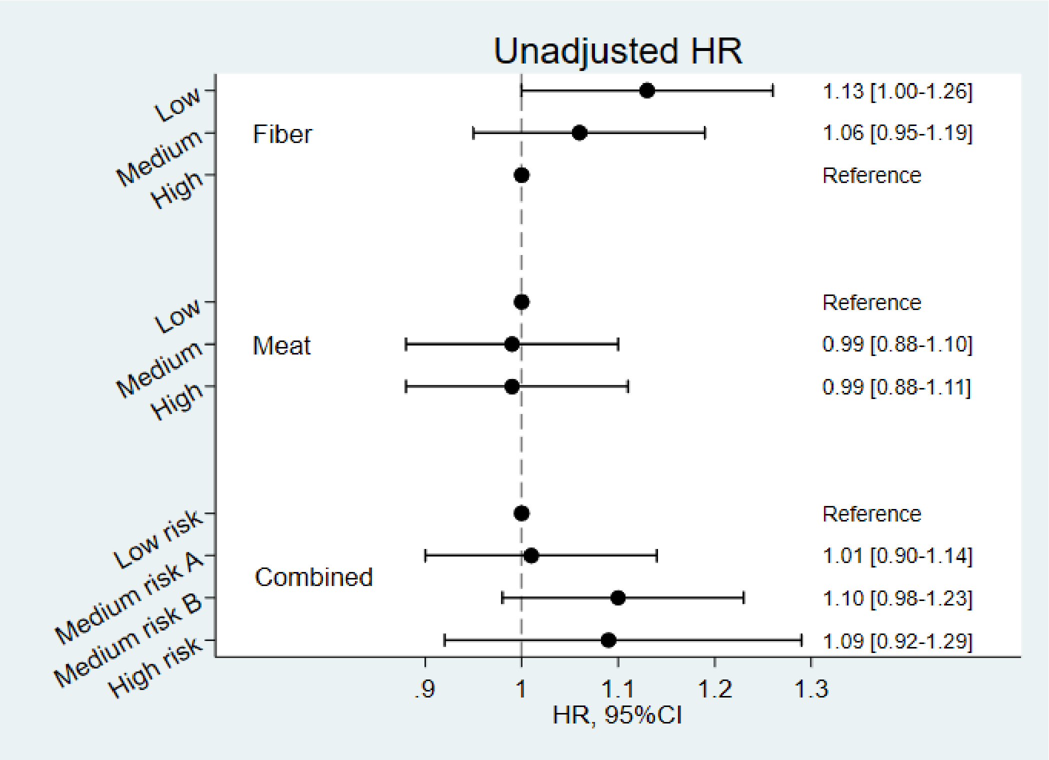

Combined exposure: Low risk: low/medium intake of meat and medium/high intake of fibres; Medium risk A: high intake of meat and medium/high intake of fibres; Medium risk B low/medium intake of meat and low intake of fibres; High risk: high intake of meat and low intake of fibres.
